# Genotypically Different Clones of *Staphylococcus aureus* Are Diverse in the Antimicrobial Susceptibility Patterns and Biofilm Formations

**DOI:** 10.1155/2013/515712

**Published:** 2013-12-25

**Authors:** Salman Sahab Atshan, Mariana Nor Shamsudin, Leslie Than Thian Lung, Zamberi Sekawi, Chong Pei Pei, Arunkumar Karunanidhi, Jayakayatri Jeevajothi Nathan, Alreshidi Mateg Ali, Ehsanollah Ghaznavi-Rad, Salwa A. Abduljaleel, Rukman Awang Hamat

**Affiliations:** ^1^Laboratory of Medical Microbiology and Parasitology, Faculty of Medicine and Health Science, Universiti Putra Malaysia, 43400 Serdang, Selangor, Malaysia; ^2^Department of Medical Microbiology, Basrah University, Basrah, Iraq; ^3^Laboratory of Marine Science and Aquaculture, Institute of Bioscience, Universiti Putra Malaysia, 43400 Serdang, Selangor, Malaysia; ^4^Department of Biomedical Sciences, Faculty of Medicine and Health Science, Universiti Putra Malaysia, 43400 Serdang, Selangor, Malaysia; ^5^Department of Biomedical Sciences, Faculty of Medicine and Health Science, Al Bukacyriyah, Saudi Arabia; ^6^Department of Microbiology and Immunology, Arak University of Medical Sciences, Arak, Iran

## Abstract

This study evaluated whether genotypically different clinical isolates of *S. aureus* have similar susceptibilities to individual antibiotics. It further aims to check the impact of biofilm on the *in vitro* activity of vancomycin, daptomycin, linezolid, and tigecycline against *S. aureus* clones. The study used a total of 60 different clinical MSSA and MRSA isolates. Susceptibilities were performed in planktonic cultures by macrobroth dilution and epsilon-test (*E test*) system. Biofilm production was determined using an adherent plate assay. The efficacy of antimicrobial activities against biofilms formation was checked using confocal laser scanning microscopy (CLSM). The study found that similar and different *spa*, MLST, and SCC*mec* types displayed high variation in their susceptibilities to antibiotics with tigecycline and daptomycin being the most effective. The biofilms were found resistant to high concentrations of most antibiotics tested with daptomycin being the most effective drug used in adhesive biofilms. A considerable difference exists among similar and various clone types against antibiotics tested. This variation could have contributed to the degree of virulence even within the same clonal genotype and enhanced heterogeneity in the infection potential. Thus, the development of a rapid and precise identification profile for each clone in human infections is important.

## 1. Introduction

*Staphylococcus aureus* is an important nosocomial and community-acquired pathogen for which few existing antibiotics are efficacious [1]. Modern MRSA has evolved from several successful clonal lineages of MSSA strains via acquisition of a mobile genetic element called staphylococcal cassette chromosome *mec *(SCC*mec*) [2]. Both methicillin—sensitive and—resistant *S. aureus* (MSSA and MRSA) are considered to have different genetic characteristics and the predominant genotypes differ geographically [3]. In the United States, ST36 and ST30 strains were epidemic in hospital and community settings [4]. After 2000, the USA300 clone (carrying the SCC*mec* IV and PVL loci) was dominant, emerging worldwide [5]. In Malaysia, most of the hospital-acquired MRSA strains were of MLST sequence type ST239, belonging to clonal cluster 8 (CC8) [6]. Whereas the most common MSSA clones circulating in Malaysia were 98 different *spa* types identified, corresponding to 8 different *spa* clonal clusters (*spa*-CCs) and majority of these isolates are multidrug resistant strains [7]. However, recent studies on antimicrobial susceptibilities have reported that even a small increase in MICs below the susceptibility range could also change the clinical efficacy of the certain drugs [8]. Standard conventional microdilution and disc diffusion methods are insufficient in identifying strains with decreased susceptibilities to drugs from those that are susceptible [9]. Therefore, the aim of the present study was to assess the *in vitro* antimicrobial susceptibilities against genetically diverse staphylococcal clones in Malaysia. The minimum biofilm reduction concentrations (MBRCs) of various antibiotics were also measured for the certain of strong biofilm positive MRSA isolates. The objectives outlined in the present study are to provide quantitative data for clinicians to improve the management of treated for the patients infected with these clones.

## 2. Materials and Methods

### 2.1. Bacterial Strains

A total of 60 clinical isolates of *S. aureus* which included 30 MRSA isolates associated with six major sequence types and 30 MSSA isolates associated with 20 sequence types were selected for this study (Table 1). The isolates were collected from Hospital Kuala Lumpur, the largest Malaysian public hospital during 2009-2010. Detailed molecular characteristics of these isolates as different clones using staphylococcal cassette chromosome *mec* (SCC*mec*) typing, staphylococcal surface protein A (*spa*) typing, and multilocus sequence typing (MLST) were previously confirmed from our laboratory by Ghaznavi-Rad et al. [6] and Ghasemzadeh-Moghaddama et al. [7]. Isolates received from lab members in the form of stock cultures were stored at −80°C in Luria-Bertani broth supplemented with 20% glycerol and thawed whenever required during the experiment. MSSA ATCC 25923 (susceptibility control) and MRSA ATCC 43300 (*mec*A positive control) were used as quality controls in present study.

### 2.2. Planktonic Susceptibility Testing

#### 2.2.1. MICs Determination

The MIC is defined as the lowest concentration (maximum dilution) of antimicrobial that will inhibit the visible growth of microorganisms after overnight incubation [10]. The MICs of the five antimicrobial agents (vancomycin, amoxicillin/clavulanic acid, daptomycin, linezolid, and tigecycline) were determined simultaneously using *E* test strips (BioMérieux SA, France) according to manufacturer's recommendations included in the packaging inserts. The system comprise a predefined antibiotic gradient ranging from 0.016 to 256 *μ*g/mL and bacterial inoculum equivalent to a turbidity of a 0.5 McFarland standard was inoculated onto Mueller-Hinton agar plates (Merck) by lawn culture. Appropriate *E* test strips were carefully placed at the center of the MHA plates and incubated at 35°C for 24 h in an incubator. Isolates were categorized based on their breakpoints for resistance according to the recommendations by Clinical and Laboratory Standards Institute (CLSI) [11].

#### 2.2.2. MBCs Determination

The MBCs were determined for each different clone types in duplicates by a macrodilution technique with Mueller-Hinton broth for vancomycin, amoxicillin/clavulanic acid, linezolid, and tigecycline according to Clinical and Laboratory Standards Institute guidelines for broth microdilution susceptibility testing [11]. Mueller-Hinton broth supplemented with calcium at 75 mg/liter (physiological ionized Ca^2+^ concentration) and magnesium at 12.5 mg/liter (SMHB-PCA) was always used for macrodilution susceptibility testing of daptomycin. The MBC was defined by previously published method [12]. Briefly, in wells where there was no visible growth (no turbidity) after overnight incubation, 100 *μ*L was subcultured to MHA and the agar plates were incubated at 35°C for colony count. MBC was defined as the highest dilution showing ≥ 99.9% kill after 24 h of incubation. Five antistaphylococcal antibiotics were purchased commercially from (BIORON, Malaysia), representing agents from the glycopeptide, *β*-lactam, lipopeptide, oxazolidinones, and glycylcycline classes. Stock solutions of this antibiotic were kept frozen at −20°C.

### 2.3. Biofilm Susceptibility Testing

In this study, 6 distinct MRSA clones were selected from our previous study and well known for their ability to form stable biofilms (Table 2) [13]. The minimum biofilm reduction concentrations (MBRCs) were determined using an adaptation of a biofilm susceptibility testing method with minor modifications [12, 14]. Briefly, isolates grown for 18 h were resuspended and diluted 1 : 100 in BHI broth (supplemented 1% glucose), and 1 mL aliquots were placed into each row of a 6-well flat-bottom microtiter plate (Nunclon; Nunc), covered with a lid, and incubated for 48 h. To remove nonadherent cells, biofilms were washed 3 times with sterile ddH_2_O and exposed to fresh serial dilution of antibiotic concentrations. Antibiotics were prepared from stock depending on the concentration to be used and prepared separately on a new flat-bottom plate containing MHB to start serial dilutions of antibiotics in 1 mL of MHB per well making the range of antibiotic concentrations start with 1 : 1 per milliliter. The concentration ranges for tested antibiotic agents were as follows: vancomycin, 8–512 *μ*g/mL; daptomycin, 2–256 *μ*g/mL; linezolid, 4–512 *μ*g/mL, and tigecycline, 2–256 *μ*g/mL. One ml of media containing the antibiotic dilutions was transferred to biofilm grown in the well of a 6-microtiter plate and incubated for next 24 h at 35°C. After incubation, the lid was removed and the wells were examined for evidence of turbidity. MBRCs were determined as the last well showing no turbidity after incubation.

### 2.4. Visualization of Antibacterial Activity

For confocal laser scanning microscopy (CLSM), the cells were grown until the stage of biofilm maturation (48 h incubation) and were directly treated with the 4 antibiotics. The following antibiotic concentrations were used for the biofilm experiments based on 2x MBRCs: 512 *μ*g/mL vancomycin, 128 *μ*g/mL daptomycin, 1024 linezolid, and 256 *μ*g/mL tigecycline. After change of new culture plate containing antibiotics, the plates were incubated at 35°C for 24 h. The Live/Dead Baclight Bacterial Viability kit (Invitrogen, Paisley, UK) was adapted system according to the manufacturer's instructions. Biofilm was stained for 5 min with 1 : 1000 dilution solutions (in 0.9% NaCl) of SYTO9 for live organisms and with propidium iodide for dead organisms. The microscopic observation and image acquisitions were performed with LSMFV1000 (Olympus, Japan). The images sizes were seen with 320 × 320 (pixel) and 100x/1.4 objective lens. The total number of organisms in a biofilm was calculated by determining the ratio of killed cells to the total cells with the mean value on 3 images and standard deviation. The images were obtained using the “FLUOVIEW FV1000” software package version 3.1.2.2 (Olympus, Japan).

### 2.5. Interpretation of Data

Results for antibiotic susceptibilities were expressed in terms of MIC_50_ and MIC_90_ (MIC for 50% and 90% of the planktonic bacteria), MBC_50_ and MBC_90_ (MBC for 50% and 90% of the planktonic bacteria), and MBEC_50_ and MBEC_90_ (MIC for 50% and 90% of the biofilm bacteria). The Chi-square test was used to evaluate the statistical significance of differences in the results. A *P* value of <0.05 was considered statistically significant.

## 3. Results

### 3.1. Planktonic Susceptibility Testing

#### 3.1.1. MICs Determination

**Figure 1:**
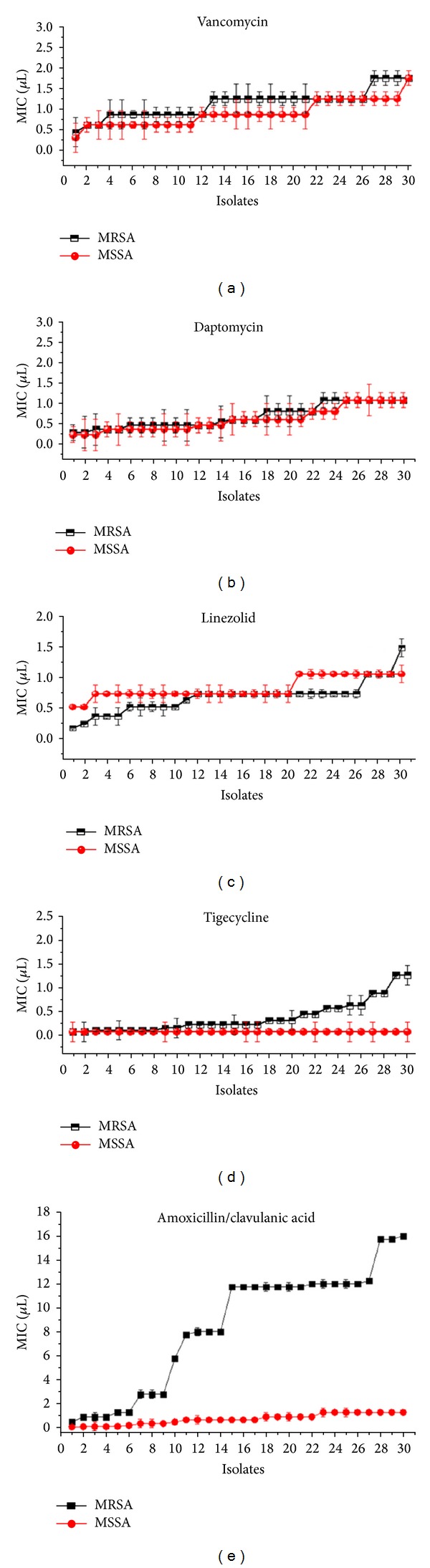
Comparing the ranges of MIC values of vancomycin, daptomycin, linezolid, tigecycline, and amoxicillin/clavulanic acid against 30 MRSA and 30 MSSA isolates.

All different clones examined were susceptible to 5 antibiotics, except 26.66 and 63.33% of MRSA that is nonsusceptible to tigecycline and amoxicillin/clavulanic acid, respectively. Although a considerable significant difference in MIC values exists among similar and various clone types of *S. aureus*, tigecycline had the lowest MIC_90_ values compared to all agents tested (Table 3). However, the MIC ranges for majority of the MRSA isolates were found to be significantly higher (*P* < 0.05) compared to the MICs for MSSA isolates (see Figure 1 and Figures S1, A, B, C, D, and E available online at http://dx.doi.org/10.1155/2013/515712).

The majority of resistant clones to the amoxicillin/clavulanic acid were found to belong to MRSA ST239-CC8 and ST22-CC22 compared to other MRSA STs (*P* < 0.05), whereas, the majority of the tigecycline resistant clones belonged to ST239-CC8-t421, ST188-CC1-t189, ST1-CC1-t127, ST1283-CC8-t037, and ST7-CC7-t091. Vancomycin sensitive MRSA isolates were found belonged to ST188-CC1 with very low MICs (*P* value < 0.05), while MRSA ST1283-CC8 showed the highest MIC for vancomycin compared to other STs. Twenty STs of MSSA clones were found to be susceptible to all antibiotics tested at different MIC values, with tigecycline being the highest degree of similarity in MIC values (see Figures S2 A and B). The MICs of control strains were found within the expected ranges (Figures S3 A, B, C, D, and E).

#### 3.1.2. MBCs Determination

**Figure 2:**
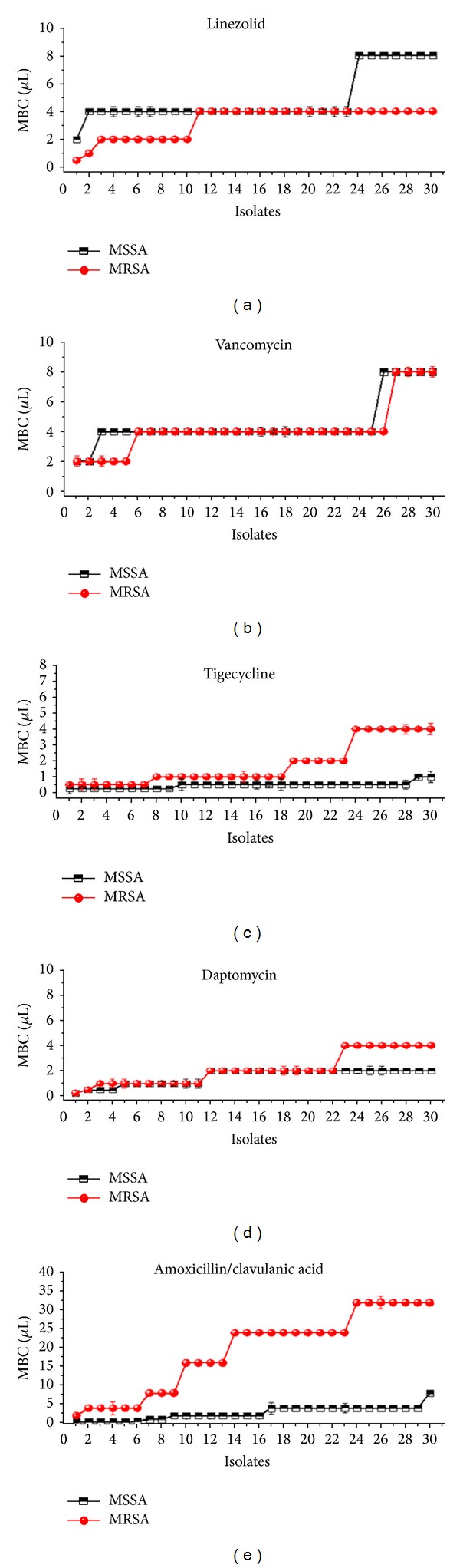
Comparing the ranges of MBC values of vancomycin, daptomycin, linezolid, tigecycline, and amoxicillin/clavulanic acid against 30 MRSA and 30 MSSA isolates.

The *in vitro* MBCs activities range, MBC50 and MBC90 (*µ*g/mL), against various antibiotics tested were found different among clone types of *S. aureus* (Table 4). Tigecycline had the lowest MBCs among the antibiotics tested, followed by daptomycin. However, the linezolid had the highest MBCs among the antibiotics tested against MSSA clones, followed by vancomycin and amoxicillin/clavulanic acid. While, amoxicillin/clavulanic acid had the highest MBCs among the antibiotics tested against MRSA clones followed by vancomycin and linezolid. The MBCs means and range values of MRSA clones were more often increased in most groups of antimicrobial agents, compared to MBCs range values of MSSA clones with positive correlation, but were statistically significant (*P* < 0.05) as illustrated in Figure 2 and Figures S4 A, B, C, D, and E.

### 3.2. Biofilm Susceptibility Testing

The MBRCs after *in vitro* biofilm formation for 6 MRSA clones were determined and are listed in Table 5. The MBRCs for vancomycin, daptomycin, linezolid, and tigecycline were overall greater than the CLSI-defined planktonic MIC breakpoint for resistance. Daptomycin and tigecycline all exhibited broad MBRC ranges. The daptomycin MBRC ranges (16–64 *μ*g/mL) and tigecycline MBRC ranges (32–128 *μ*g/mL) of 6 isolates were overall much lower than other MBRC ranges.

### 3.3. Efficacies of Antibiotics on Adherent Biofilms

**Figure 3:**
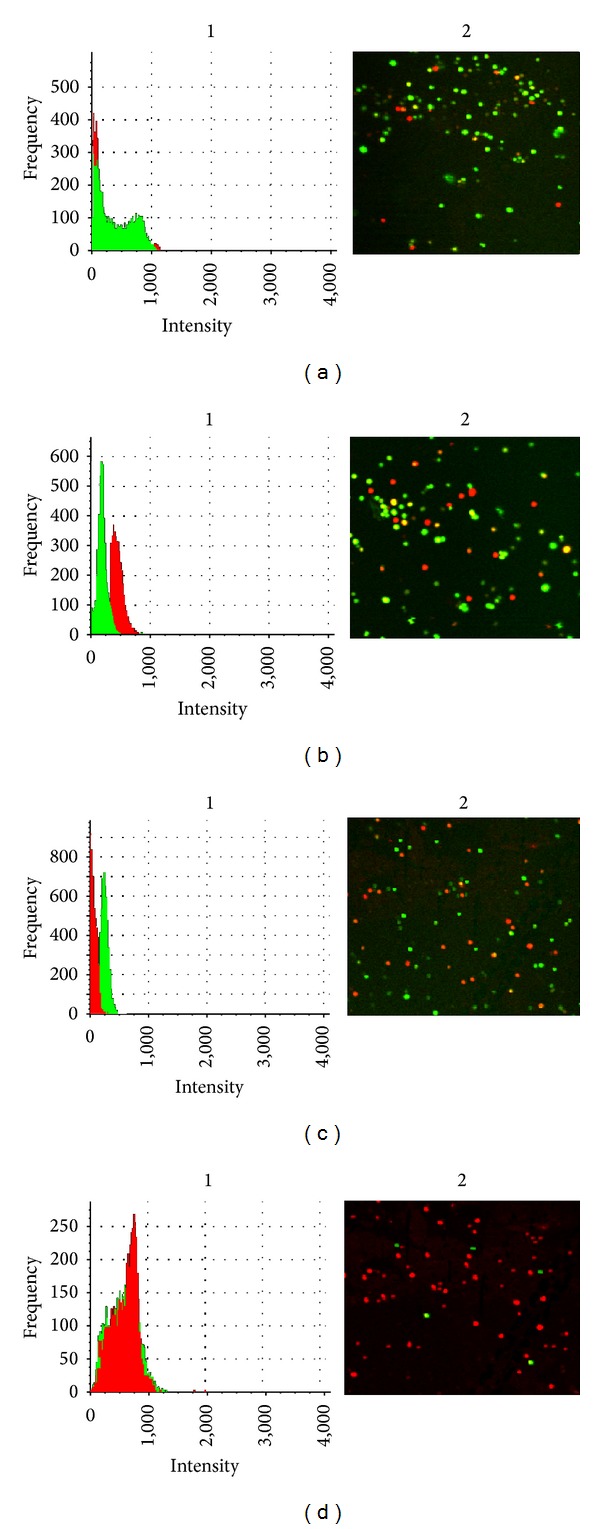
Efficacy of antibiotic treatments against biofilm cells. The cells on mature biofilms (48 h incubation) were treated with 512 *μ*g/mL vancomycin, 128 *μ*g/mL daptomycin, 1024 linezolid, and 256 *μ*g/mL tigecycline. After 24 h of the antibiotic treatments, the cells were stained with Live/Dead kit. 1 indicates the histograms of frequency and intensity of live and dead cells. 2 indicates the acquired 2D view images obtained at 1.4 objective and magnification of ×100; (a) linezolid, (b) vancomycin, (c) tigecycline, (d) daptomycin.

As shown in Figures 3(a), 3(b), 3(c), and 3(d), the remaining adherent biofilms populations differed between the 4 antibiotics. Most cells were alive following linezolid, vancomycin, tigecycline, and daptomycin treatment. Linezolid, vancomycin, and tigecycline killed 16.6 ± 2.1%, 28.5 ± 2.4%, and 55.5 ± 3.4% of the cells in mature *S. aureus* biofilms, respectively. Live/Dead staining revealed that daptomycin was the most efficient in reducing the number of biofilms, as 93 ± 2.8% of the cells were killed.

## 4. Discussion

Due to the clonal variations, there is a drastic change in the antibiotic susceptibility patterns among microbial populations. The rate of resistance varies geographically within countries depending on antibiotic policies and enforcements by infection control boards and under various clone types reporting. It is important to conduct regular antibiogram studies on frequently encountered superbugs like *S. aureus* clones, which are prone to acquire multidrug resistance. Although the number of antibiotics selected in the present study is less, the antibiograms obtained will update changes in susceptibility patterns and treatment options. The study also utilized a limited number of clones (60 clone types); however, it is the total number of nonduplicate the isolates obtained from a tertiary hospital over a one year period. Several surveillance studies on the increased prevalence of MRSA have been reported earlier [15, 16]. Very high prevalence rates of MRSA have been documented in developed countries, especially in Western Pacific regions, both in community-acquired and hospital infections [15]. According to the surveys conducted in Malaysian hospitals, the prevalence of MRSA increased from the range of 10–25% in 1985-1986 to more than 40% in 1996 [17, 18]. Of the 30 MRSA clones tested, 26.6% clones were resistant to tigecycline and 63.3% were found to be amoxicillin/clavulanic acid resistant. However, all clones of MSSA were sensitive to all the antibiotics tested (Table 3). It is not surprising as majority of the MRSA clones were resistant to amoxicillin/clavulanic acid making it ineffective against MRSA even *in vitro*, but this antibiotic showed excellent activity against MSSA clones. It is conceivable that the changes mediating reduced susceptibility to beta-lactamantibiotics in MRSA are most likely caused by an intrinsic resistance mechanism of *mec*A gene, which encodes penicillin-binding protein 2′ (PBP2′) with significantly reduced affinity for *β*-lactams [19]. Although, amoxicillin/clavulanic acid exhibits *in vitro* activity against certain MRSA clones, it is not clinically effective and MRSA clones should therefore be considered resistant. Among the six major sequence types of MRSA utilized in this study, ST22-CC22 and ST239-CC8 were found to be the highly resistant clones towards amoxicillin/clavulanic acid. Previously Ghaznavi-Rad et al. [20] utilized antibiogram and MICs of selected antibiotics, including oxacillin by *E* test for the same strain of MRSA used in this study and showed the isolates belonged to clonal complex 8 (CC8) and CC22 (ST-22) had high-level resistance to oxacillin (MIC 8–256 mg/L), while other STs showed low-level resistance to oxacillin (MIC 4–8 mg/L). However, ST22-CC22 and ST239-CC8 STs of MRSA have increased MIC values to daptomycin compared to other STs associated with low-level resistance (Figure S2, B). Ghaznavi-Rad et al. [6] also have documented that the ST22-CC22 of SCC*mec *type IVh would have recently emerged in Malaysia and the element of this clone is small in comparison to elements of other STs such as SCC*mec *type IIIA of ST239-CC8, the small size enables easier spread among *S. aureus *populations, and acquired the resistance [21, 22]. In addition, the presence of the ACME arcA gene in all ST-239 SCC*mec* type III MRSA clones tested in the present study has been previously confirmed by Ghaznavi-Rad et al. [6]. This ACME arcA contributes to the growth and survival by encoding resistance and virulence determinants that enhance clearer discrimination of predominant MRSA t037-ST239 as well as their resistantce to various classes of antibiotics [23, 24]. This may possibly reflect the prevalence of ST239-CC8 in Malaysia and in neighbouring Asian countries in recent years, which are highly resistant clones in general. Tigecycline showed excellent activity against all the 30 MSSA tested with acceptable ranges of MIC_50_ (0.07 *μ*g/mL) and MIC_90_ (0.07 *μ*g/mL). However, the MICs of tigecycline for the MRSA clones tested were found to be slightly higher than that for MSSA clones (less correlation) but statistically significant (*r*  =  −0.43; *P* < 0.05) (Figure 1 and Figure S1 (E)). These results are similar to the observations by Fluit et al. [25]. An interesting aspect of this study is the remarkable difference in resistance against tigecycline among the 6 MRSA STs tested (Figure S2, B). Of all the MRSA STs, ST22-CC22 was found to be highly susceptible to tigecycline, whereas other MRSA STs showed varying susceptibilities towards tigecycline. This discrepancy requires further investigations into the underlying the mechanisms of varying susceptibilities and resistance. Vancomycin remains the choice of therapy for serious MRSA infections, but its efficacy is inferior to that of antistaphylococcal penicillins against MSSA [26]. The MICs of vancomycin for MRSA clones observed in the present study appear to be slightly higher than those for MSSA clones, although the correlation is relatively strong and statistically significant (*r*  =  0.85; *P* < 0.05) (Figure 1 and Figure S1(A)). This slight increase in MICs with MRSA clones are a worrying finding. An increase in vancomycin MIC, even within the susceptible range, has raised the risk of treatment failure in cases of MRSA infection [27]. All the 60 clone types were susceptible to daptomycin and linezolid antibiotics and this is similar to the observations by Perry et al. [28]. High proportion of MRSA clones had a little variation in MICs for daptomycin and linezolid, which is not observed in MSSA clones (Figure 1 and Figures S1 (C) and (D)). This slight increase in daptomycin MICs for MRSA might be due to the thickening of cell wall and not because of the intrinsic resistance mechanism [29]. Based on the results of the current study, the most effective therapeutic options for MRSA infections identified are daptomycin and tigecycline for MSSA infections.

We also analyzed the bactericidal activities of various antimicrobial agents against different clone types of MRSA and MSSA based on the eradication of cells in planktonic. Bactericidal activity appears to be necessary for clinical efficacy in certain circumstances, for example, endocarditis, meningitis, osteomyelitis, and severe infections involving neutropenic patients [30]. In the present study, although the MBC values in some cases exceeded the highest drug concentration tested, daptomycin and tigecycline had the lowest MBC90 values compared to all agents tested with MBC ranges being slightly different between MSSA and MRSA clones (Table 4). The MBC ranges of vancomycin and daptomycin were slightly similar for all clones of MSSA and MRSA, while the MBC ranges and means of linezolid, tigecycline, and amoxicillin/clavulanic acid had significant different bactericidal activities in both MSSA and MRSA clones (Figure 2 and Figures S4 A, B, C, D and E). A recent study revealed that daptomycin showed noninferiority, as compared with the standard regimens, for treating both MSSA and MRSA bacteraemia and endocarditis [31]. Our results showed that daptomycin and tigecycline had the most potent *in vitro* activity among the agents studied; therefore it has been suggested to be used for *S. aureus* clone types infections.

The ability of *S. aureus* to form biofilms contributes to antibiotic resistance, and frequently more MRSA strains are causing biofilm-associated infections [32, 33]. Further, biofilm susceptibility testing gives quantitative data, which are not obtainable with the Kirby-Bauer method. These quantitative data are useful in predicting the levels of antibiotics that must be attained to assure inhibition or killing of biofilm. Few studies have investigated the abilities of different MRSA clones in hospitals and communities to form biofilms. In Malaysian MRSA clones, there was no data available to provide biofilm information on antibiotic selection in infected patients. During *in vitro* biofilms, all clones induced biofilms were resistant to drug concentrations 16 to 512 times greater than the planktonic susceptibility breakpoints, with daptomycin and tigecycline being the most effective drugs used in adhesive biofilms. Based on microscopy studies confirming that daptomycin may be the treatment of choice to prevent biofilm regrowth at lower concentrations than could the other drugs (Figure 3); this was in agreement with another study [34], which cited that the daptomycin and tigecycline activities in biofilms were better than those of the other antibiotics used.

## 5. Conclusion

The present study shows a considerable difference exists among similar and various clone types of *S. aureus* with significant variation in antibiotic susceptibility being observed. Thus, the development of a rapid and precise identification profile for each clone in human infections is important in order to prescribe the correct antibiotic and reduce empirical treatment. In addition, clones of *S. aureus* that are positive for biofilms were increased in resistance to most of antibiotics with daptomycin and tigecycline being lower overall compared to those of other antibiotics in planktonic and attached biofilms. These *in vitro* data against various clones could provide a basis for the use of antibiotics in the treatment of biofilm staphylococcal infections.

## Supplementary Material

Figure S1 (A): Linear regression analysis of MIC values for vancomycin, against both MRSA and MSSA isolates. (B): Linear regression analysis of MIC values for *amoxicillin/clavulanic acid* against both MRSA and MSSA isolates. (C): Linear regression analysis of MIC values for *daptomycin* against both MRSA and MSSA isolates. (D): Linear regression analysis of MIC values for linezolid against both MRSA and MSSA isolates. (E): Linear regression analysis of MIC values for tigecycline against both MRSA and MSSA isolates.
Figure S2: *In-vitro* activity of vancomycin, *amoxicillin/clavulanic acid*, linezolid, daptomycin and tigecycline against 20 sequence types of 30 MSSA(A) and 6 major sequence types of 30 MRSA isolates (B) prevalence in Malaysia.
Figure S3 (A): Vancomycin read at endpoint of MIC 1.00 and 0.75 **μ**g/mL against MSSA-ATCC25643 and MRSA-ATCC43300 reference strain, respectively, using E test system. (B): *Amoxicillin/clavulanic acid* at endpoint of MIC 0.47 and 1.0 **μ**g/mL against MSSA-ATCC25643 and MRSA-ATCC43300 reference strain, respectively, using E test system. (C): Linzolid read at endpoint of MIC 5.0 and 0.19 **μ**g/mL against MSSA-ATCC25643 and MRSA-ATCC43300 reference strain, respectively, using E test system. (D): Daptomycin read at endpoint of MIC 0.64 and 0.64 **μ**g/mL against MSSA-ATCC25643 and MRSA-ATCC43300 reference strain, respectively, using E test system. (E): Tigecycline read at endpoint of MIC 0.64 and 0.94 **μ**g/mL against MSSA-ATCC25643 and MRSA-ATCC43300 reference strain, respective, using E test system.
Figure S4 (A): Linear regression analysis of MBC values for Vancomycin, against both MRSA and MSSA isolates. (B): Linear regression analysis of MBC values for linezolid against both MRSA and MSSA isolates. (C): Linear regression analysis of MBC values for tigecycline against both MRSA and MSSA isolates. (D): Linear regression analysis of MBC values for *daptomycine* against both MRSA and MSSA isolates. (E): Linear regression analysis of MBC values for *amoxicillin/clavulanic acid* against both MRSA and MSSA isolates.

## Figures and Tables

**Table 1:** Characteristics of isolates used in this study.

MLST sequence type	SCC*mec * type	No. of isolates	Source of isolates
ST 239-CC8	IIIA	13-MRSA	Pus (4), sputum (3), wound (3), blood (1), abscess (1), ^b^C.S.F (1)
ST22-CC22	IVh	5-MRSA	Blood (4), pus (1)
ST188-CC1	V	3-MRSA	Pus (2), wound (1)
ST1-CC1	V	3-MRSA	Pus (2), wound (1)
ST1283-CC8	IIIA	3-MRSA	Medical devices (1), blood (1), wound (1)
ST7-CC7	V	3-MRSA	Pus (2), blood (1)
ST5-CC5	—	4-MSSA	Tracheal aspirate (1), ^c^swab (1), urine (1), tissue (1)
ST121-CC121	—	4-MSSA	Swab (3), abscess (1)
ST88-CC88	—	2-MSSA	Swab (2)
ST45-CC45	—	2-MSSA	Swab (2)
ST8-CC8	—	2-MSSA	Swab (2)
ST15-CC15	—	2-MSSA	C.S.F (1), urine (1)
^ a^Different ST types	—	14-MSSA	Swab (7), tissue (2), pus (2), abscess (1), C.S.F (1), tracheal aspirate (1)

^a^Major STs of MSSA recovered as single isolates including ST188-singleton, ST152-CC8, ST80-CC80, ST1-CC1, ST12-CC12, ST20-singleton, ST361-singleton, ST1153-singleton, ST769-CC1, ST508-CC45, ST833-singleton, ST9-CC97, ST1050-CC25, and ST15-CC5. ^b^C.S.F: cerebrospinal fluid. ^c^Swab: specimen collected from haematoma, eyes, axilla, skin, breast, face, foot, and ear.

**Table 2:** The biofilm positive MRSA isolates used in this study.

Isolates	*spa* types	MLST		SCC*mec *	Biofilm forming	PCR *ica*ABCD	MBC (*μ*g/mL)			
		ST	CC				Vanc.	Lin.	Tig.	Dap.
1 (MRSA)/527	t037	ST-239	CC8	IIIA	++++	+	4	2	1	2
2 (MRSA)/524	t037	ST-239	CC8	IIIA	++++	+	2	2	1	4
3 (MRSA)/5	t421	ST-239	CC8	IIIA	++++	+	4	2	0.5	1
4 (MRSA)/526	t421	ST-239	CC8	IIIA	++++	+	4	4	2	2
5 (MRSA)/418	t127	ST-1	CC1	V	++++	+	8	4	4	1
6 (MRSA)/404	t127	ST-1	CC1	V	++++	+	4	4	0.5	0.5

Vanc: vancomycin, Lin: linezolid, Tig: tigecycline, Dap: daptomycin.

**Table 3:** *In vitro* MICs comparing activities of five antimicrobial agents against 30 MSSA and 30 MRSA different isolates isolated from a clinical setting in largest Malaysian public hospital.

Organism tested (no. of isolates) and antibiotic agent	MIC (*μ*g/mL)			% Susceptibility	
	Range	50%	90%	S	R
MSSA (30)					
Vancomycin	0.31–1.75	0.62	0.87	100	0
Linezolid	0.62–1.25	0.87	0.87	100	0
Tigecycline	0.07-0.07	0.07	0.07	100	0
Daptomycin	0.10–0.87	0.22	0.44	100	0
Amox.*/*clav.	0.03–1.25	0.62	1.25	100	0
MRSA (30)					
Vancomycin	0.44–1.75	0.87	1.25	100	0
Linezolid	0.22–1.75	0.87	0.87	100	0
Tigecycline	0.07–1.25	0.22	0.22	73.33	26.66
Daptomycin	0.15–0.87	0.31	0.87	100	0
Amox.*/*clav.	0.44–16	12	12	36.66	63.33

Amox.*/*clav: amoxicillin*/*clavulanic acid; S: sensitive; R: resistant; MIC: minimum inhibition concentration; MSSA: methicillin-susceptible *S. aureus*; MRSA: methicillin-resistant *S. aureus.*

**Table 4:** *In vitro* MBCs comparing activities of 5 antimicrobial agents against 30 MSSA and 30 MRSA different isolates isolated from a clinical setting in largest Malaysian public hospital.

Organism tested (no. of isolates) and antibiotic agent	MBC (**μ**g/mL)		
	Range	50%	90%
MSSA (30)			
Vancomycin	2–8	4	4
Linezolid	2–8	4	4
Tigecycline	0.25–1	0.25	0.5
Daptomycin	0.25–2	2	2
Amox./clav.	0.25–8	2	4
MRSA (30)			
Vancomycin	2–8	4	4
Linezolid	0.5–4	4	4
Tigecycline	0.5–4	0.5	1
Daptomycin	0.125–4	1	2
Amox./clav.	2–32	4	24

**Table 5:** Minimal biofilm reduction concentrations of different antimicrobials on six strong biofilm-forming MRSA isolates.

Isolates number	MBEC (*μ*g/mL)			
	Vancomycin	Daptomycin	Linezolid	Tigecycline
1 (MRSA)/527	128	32	256	64
2 (MRSA)/524	256	64	512	128
3 (MRSA)/5	256	64	128	128
4 (MRSA)/526	256	64	512	128
5 (MRSA)/418	64	64	512	32
6 (MRSA)/404	256	16	512	128
MBEC50	256	64	512	128
MBEC90	256	64	512	128
Range	64–256	16–64	128–512	32–128
