# Supplementary material for: Genotypically Different Clones of Staphylococcus aureus Are Diverse in the Antimicrobial Susceptibility Patterns and Biofilm Formations
Source: Biomed Res Int. 2013 Dec 25;2013:515712. doi: 10.1155/2013/515712 (PMC3886620; doi:10.1155/2013/515712)
Supplement: Supplementary file 1 — Figure S1 (A): Linear regression analysis of MIC values for vancomycin, against both MRSA and MSSA isolates. (B): Linear regression analysis of MIC values for amoxicillin/clavulanic acid against both MRSA and MSSA isolates. (C): Linear regression analysis of MIC values for daptomycin against both MRSA and MSSA isolates. (D): Linear regression analysis of MIC values for linezolid against both MRSA and MSSA isolates. (E): Linear regression analysis of MIC values for tigecycline against both MRSA and MSSA isolates. Figure S2: In-vitro activity of vancomycin, amoxicillin/clavulanic acid, linezolid, daptomycin and tigecycline against 20 sequence types of 30 MSSA(A) and 6 major sequence types of 30 MRSA isolates (B) prevalence in Malaysia. Figure S3 (A): Vancomycin read at endpoint of MIC 1.00 and 0.75 μg/mL against MSSA-ATCC25643 and MRSA-ATCC43300 reference strain, respectively, using E test system. (B): Amoxicillin/clavulanic acid at endpoint of MIC 0.47 and 1.0 μg/mL against MSSA-ATCC25643 and MRSA-ATCC43300 reference strain, respectively, using E test system. (C): Linzolid read at endpoint of MIC 5.0 and 0.19 μg/mL against MSSA-ATCC25643 and MRSA-ATCC43300 reference strain, respectively, using E test system. (D): Daptomycin read at endpoint of MIC 0.64 and 0.64 μg/mL against MSSA-ATCC25643 and MRSA-ATCC43300 reference strain, respectively, using E test system. (E): Tigecycline read at endpoint of MIC 0.64 and 0.94 μg/mL against MSSA-ATCC25643 and MRSA-ATCC43300 reference strain, respective, using E test system. Figure S4 (A): Linear regression analysis of MBC values for Vancomycin, against both MRSA and MSSA isolates. (B): Linear regression analysis of MBC values for linezolid against both MRSA and MSSA isolates. (C): Linear regression analysis of MBC values for tigecycline against both MRSA and MSSA isolates. (D): Linear regression analysis of MBC values for daptomycine against both MRSA and MSSA isolates. (E): Linear regression analysis of MBC values for amoxicilli [file 515712.f1.docx]

Supplementary materials


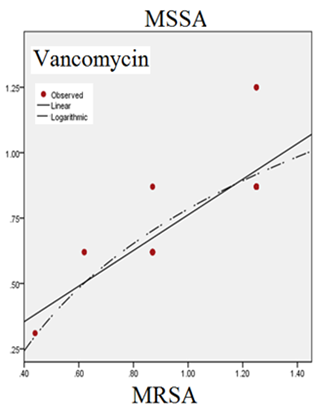


**Figure S1 (A):** Linear regression analysis of MIC values for vancomycin, against both MRSA and MSSA isolates. The MICs of vancomycin correlated strongly (statistically significantly), with those of vancomycin against both MSSA and MRSA (*r*= 0.85; *P* < 0.05).


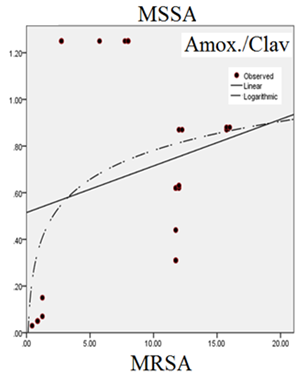


**Figure S1(B):** Linear regression analysis of MIC values for amoxicillin */*clavulanic acid against both MRSA and MSSA isolates . The MICs of amoxicillin /clavulanic acid correlated weakly with statistically significantly, with those of amoxicillin */*clavulanic acid against both MSSA and MRSA (*r* = 0.28; *P* < 0.05).


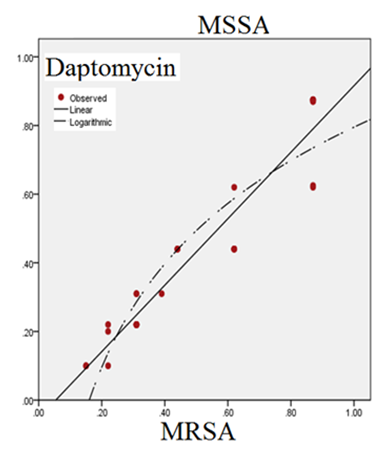


**Figure S1 (C):** Linear regression analysis of MIC values for daptomycin against both MRSA and MSSA isolates. The MICs for daptomycin showed a significant correlation with those of daptomycin against both MSSA and MRSA (r=0.95; *P* < 0.05).


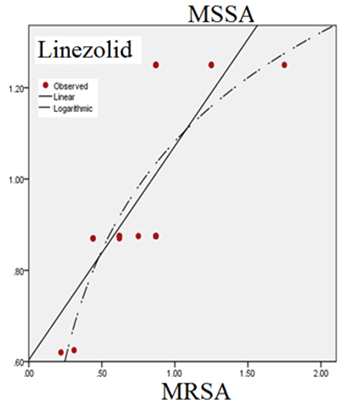


**Figure S1 (D):** Linear regression analysis of MIC values for linezolid against both MRSA and MSSA isolates. The MICs for linezolid showed a significant correlation with those of linezolid against both MSSA and MRSA (*r* = 0.70; *P* < 0.05).


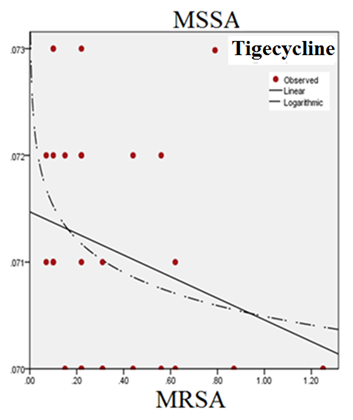


**Figure S1 (E):** Linear regression analysis of MIC values for tigecycline against both MRSA and MSSA isolates. The MICs for tigecycline showed a negative correlated (statistically significantly) with those of tigecycline against both MSSA and MRSA (*r* = ‒0.43; *P* <0.05).


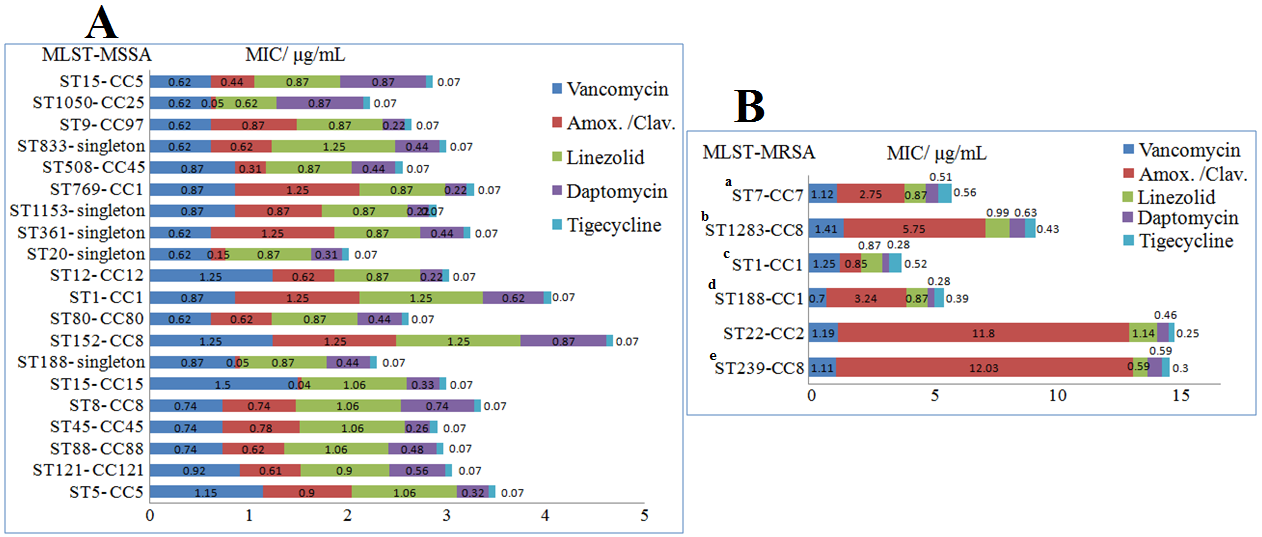


**Figure S2:** *In-vitro* activity of vancomycin, amoxicillin */*clavulanic acid*,* linezolid, daptomycin and tigecycline against 20 sequence types of 30 MSSA(A) and 6 major sequence types of 30 MRSA isolates (B) prevalence in Malaysia .^a^ indicates to 3 frequent ST7-CC7 had MIC values for tigecycline less than 0.5 μg/ml with the exception of 1 clones had resistant to tigecycline.^b^ indicates to 3 frequent ST1283-CC8 had MIC values for tigecycline less than 0.5 μg/ml with the exception of 2 clones had resistant to tigecycline. ^c^ indicates to 3 frequent ST1-CC1 had MIC values for tigecycline less than 0.5 μg/ml with the exception of 1 clones had resistant to tigecycline.^d^ indicates to 3 frequent ST188-CC1 had MIC values for tigecycline less than 0.5 μg/ml with the exception of 1 clone had resistant to tigecycline. ^e^ indicates to13 frequent ST 239-CC8 had MIC values for tigecycline less than 0.5 μg/ml with the exception of 3 clones had resistant to tigecycline with MIC value more than 0.5 μg/ml.

**
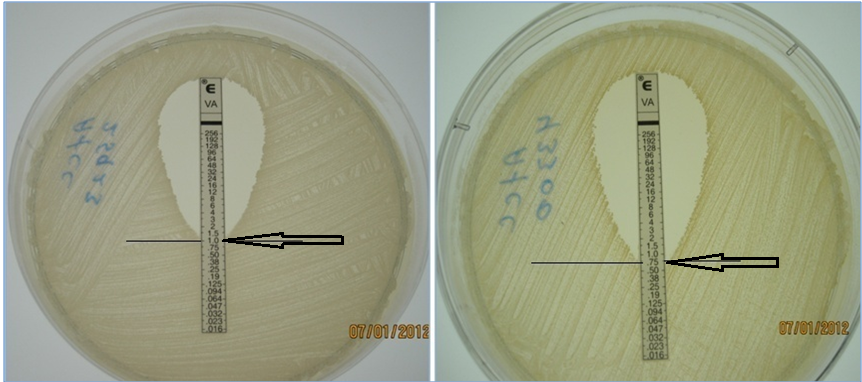
**

**Figure S3 (A):** Vancomycin read at endpoint of MIC 1.00 and 0.75 μg/mL against MSSA-ATCC25643 and MRSA-ATCC43300 reference strain, respectively, using E test system.

**
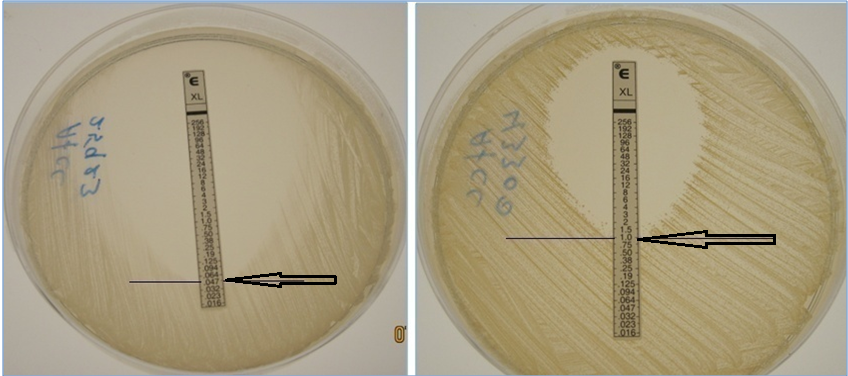
**

**Figure S3 (B):** Amoxicillin */*clavulanic acid at endpoint of MIC 0.47 and 1.0 μg/mL against MSSA-ATCC25643 and MRSA-ATCC43300 reference strain, respectively, using E test system.

**
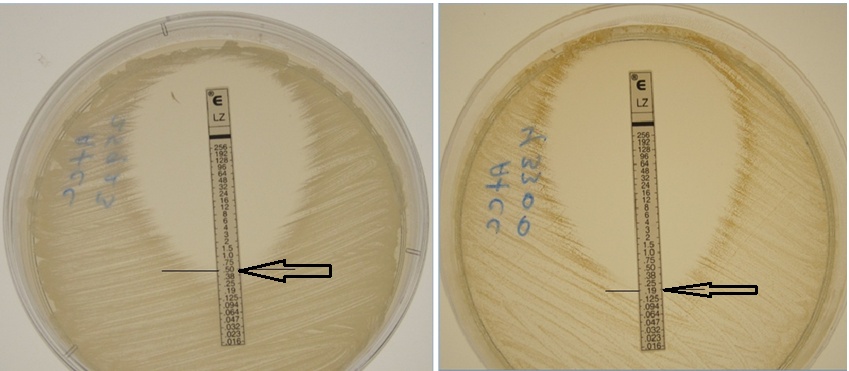
**

**Figure S3 (C):** Linzolid read at endpoint of MIC 5.0 and 0.19 μg/mL against MSSA-ATCC25643 and MRSA-ATCC43300 reference strain, respectively, using E test system.

**
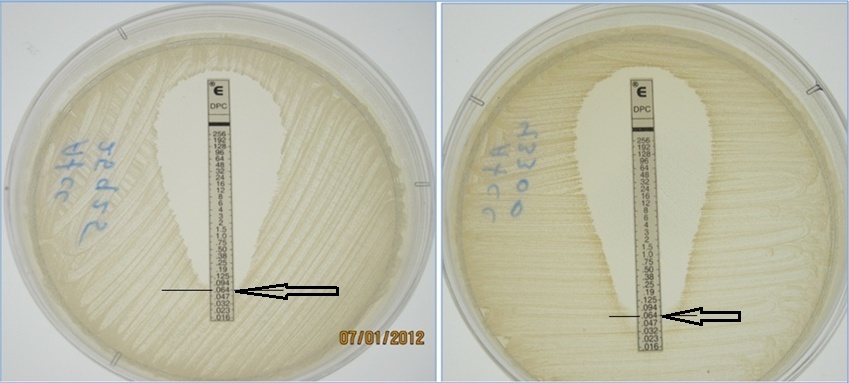
**

**Figure S3 (D):** Daptomycin read at endpoint of MIC 0.64 and 0.64 μg/mL against MSSA-ATCC25643 and MRSA-ATCC43300 reference strain, respectively, using E test system.

**
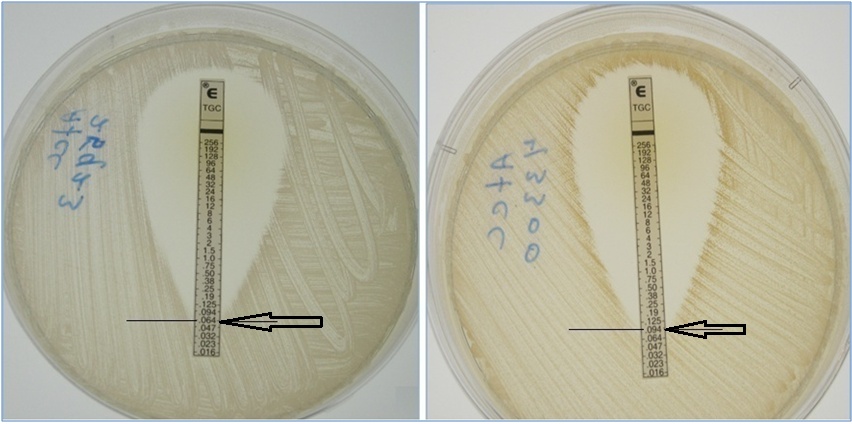
**

**Figure S3 (E):** Tigecycline read at endpoint of MIC 0.64 and 0.94 μg/mL against MSSA-ATCC25643 and MRSA-ATCC43300 reference strain, respective, using E test system.


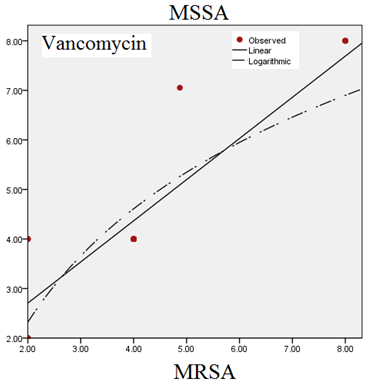


**Figure S4 (A):** Linear regression analysis of MBC values for Vancomycin, against both MRSA and MSSA isolates. The MBCs of vancomycin correlated positive (statistically significantly), with those of vancomycin against both MSSA and MRSA (*r*= 0.84; *P <*0.01).


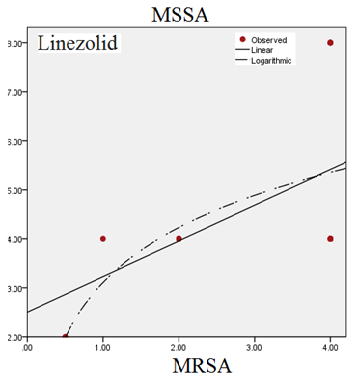


**Figure S4 (B):** Linear regression analysis of MBC values for linezolid against both MRSA and MSSA isolates. The MBCs for linezolid showed a correlated positive (statistically significantly) with those of linezolid against both MSSA and MRSA (*r* = 0.45 *P* <0.05).


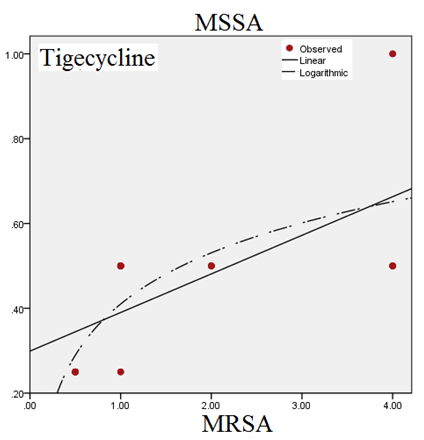


**Figure S4 (C):** Linear regression analysis of MBC values for tigecycline against both MRSA and MSSA isolates. The MBCs for tigecycline showed a correlated positive (statistically significantly) with those of tigecycline against both MSSA and MRSA (*r* = ‒0.65; *P* < 0.01).


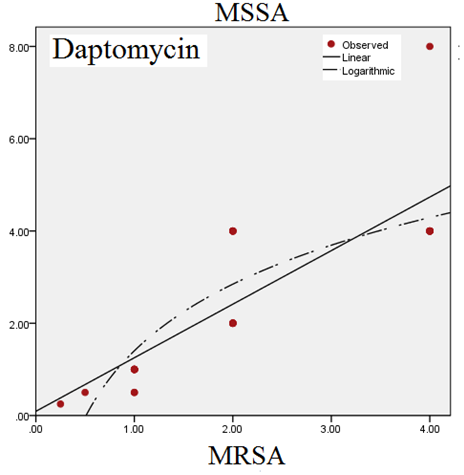


**Figure S4 (D**): Linear regression analysis of MBC values for daptomycine against both MRSA and MSSA isolates. The MBCs for daptomycin showed a correlated positive (statistically significantly) with those of daptomycin against both MSSA and MRSA (r=0.82; *P* < 0.01).


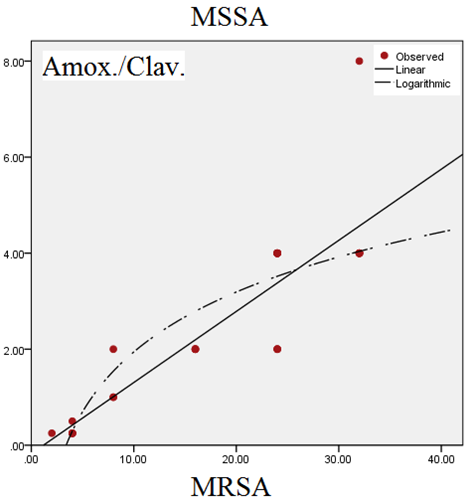


**Figure S4 (E):** Linear regression analysis of MBC values for amoxicillin */*clavulanic acid against both MRSA and MSSA isolates. The MBCs of amoxicillin */*clavulanic acid correlated positive with statistically significantly, with those of amoxicillin */*clavulanic acid against both MSSA and MRSA (*r* = 0.86; *P* < 0.01).
